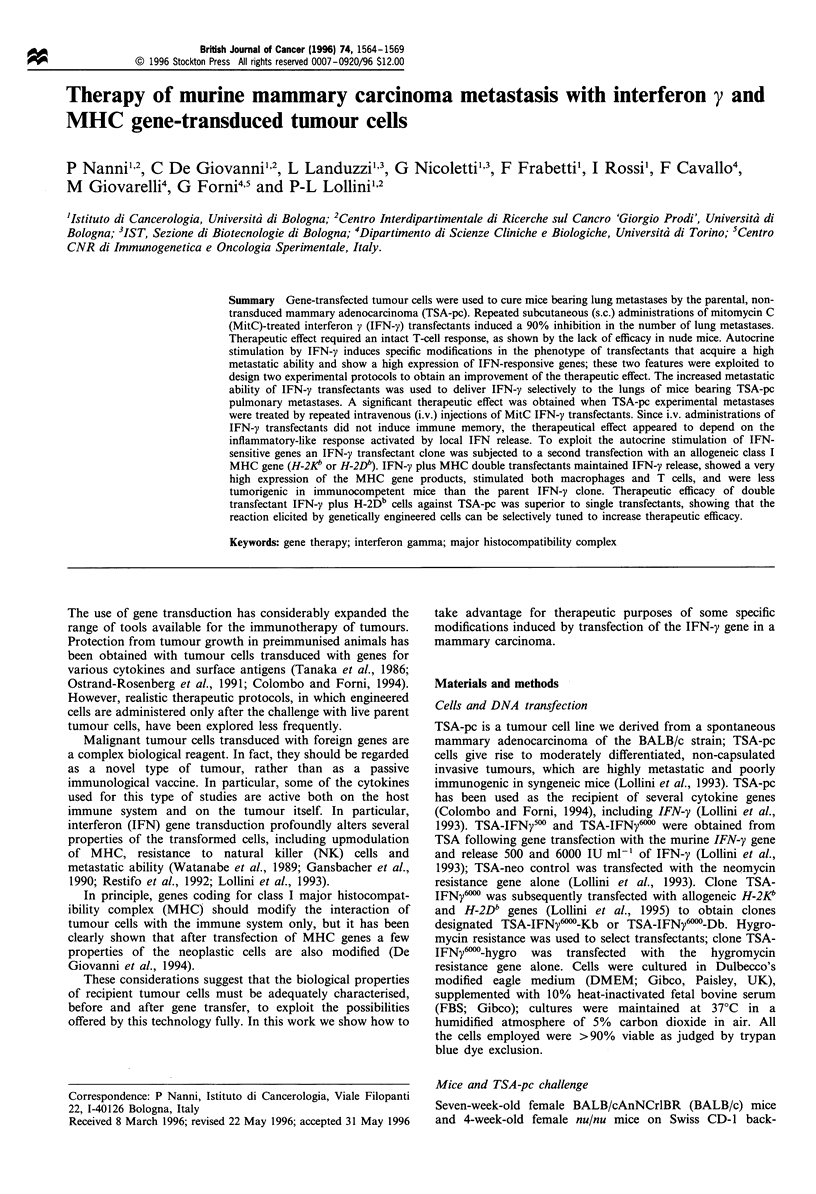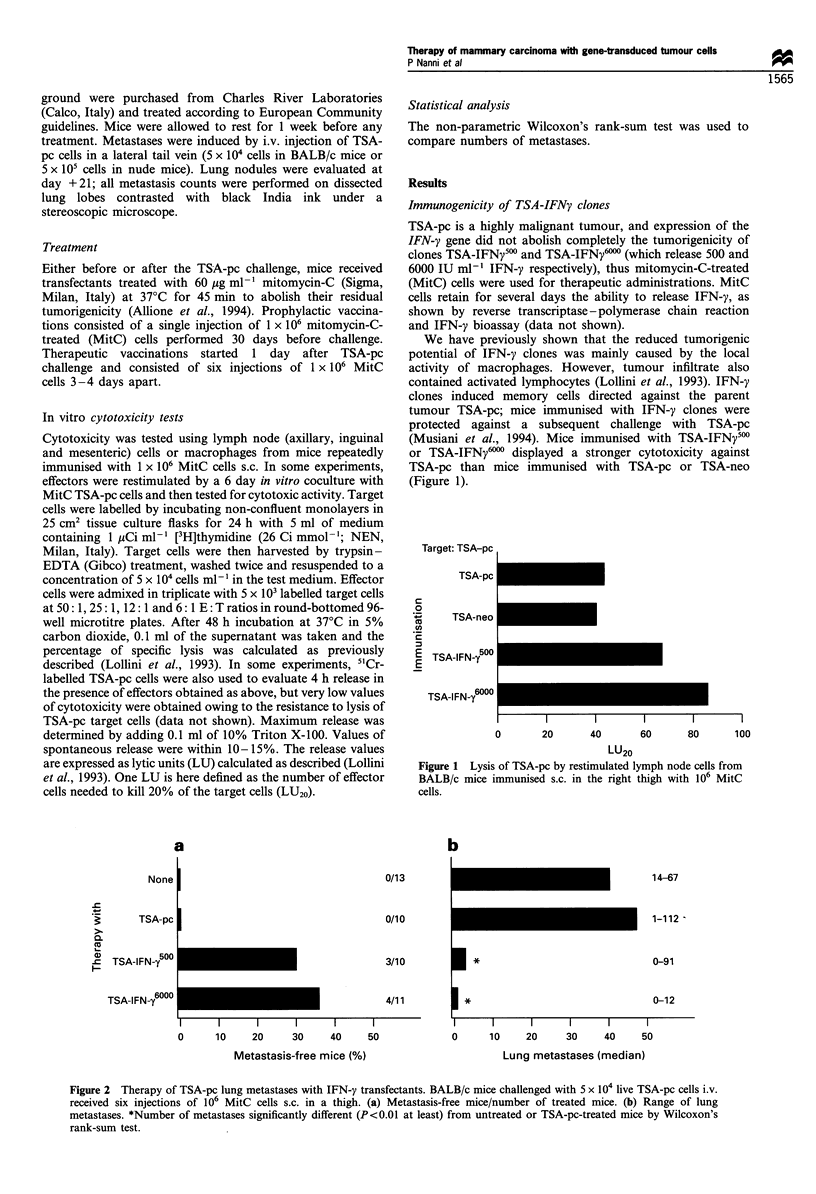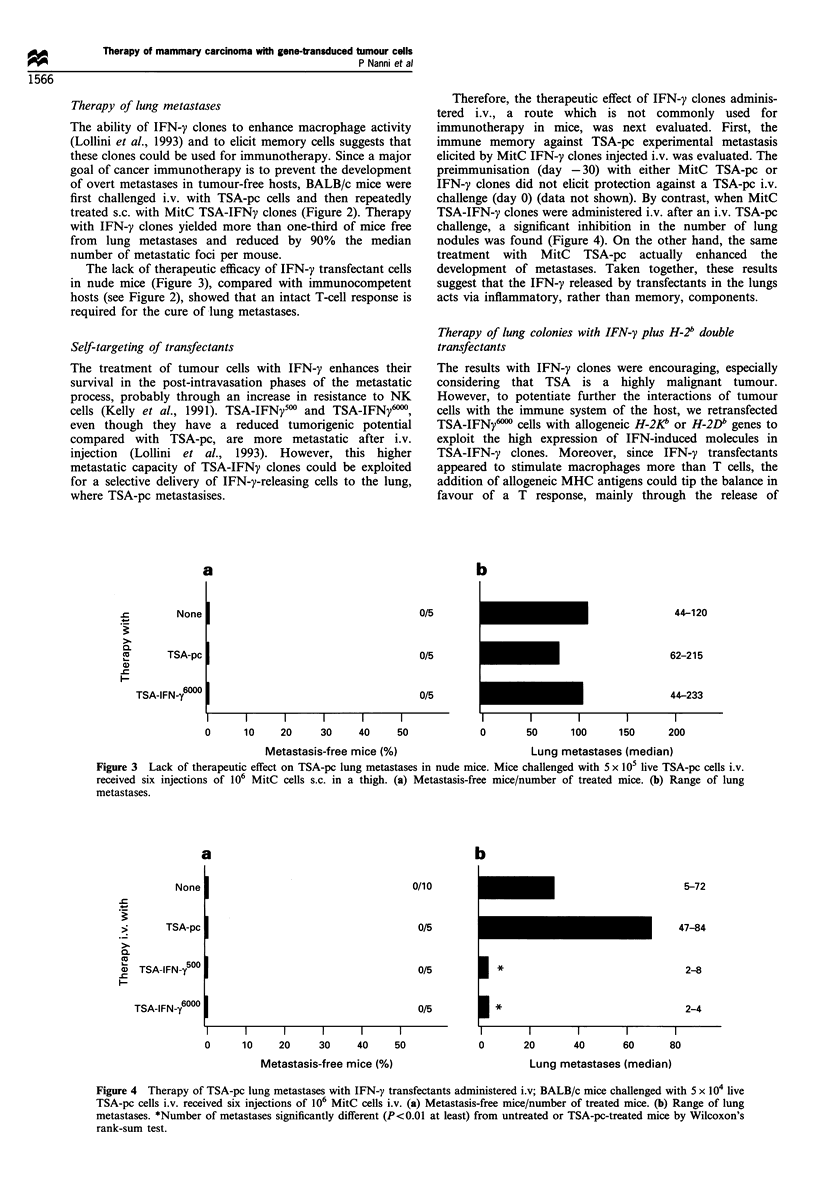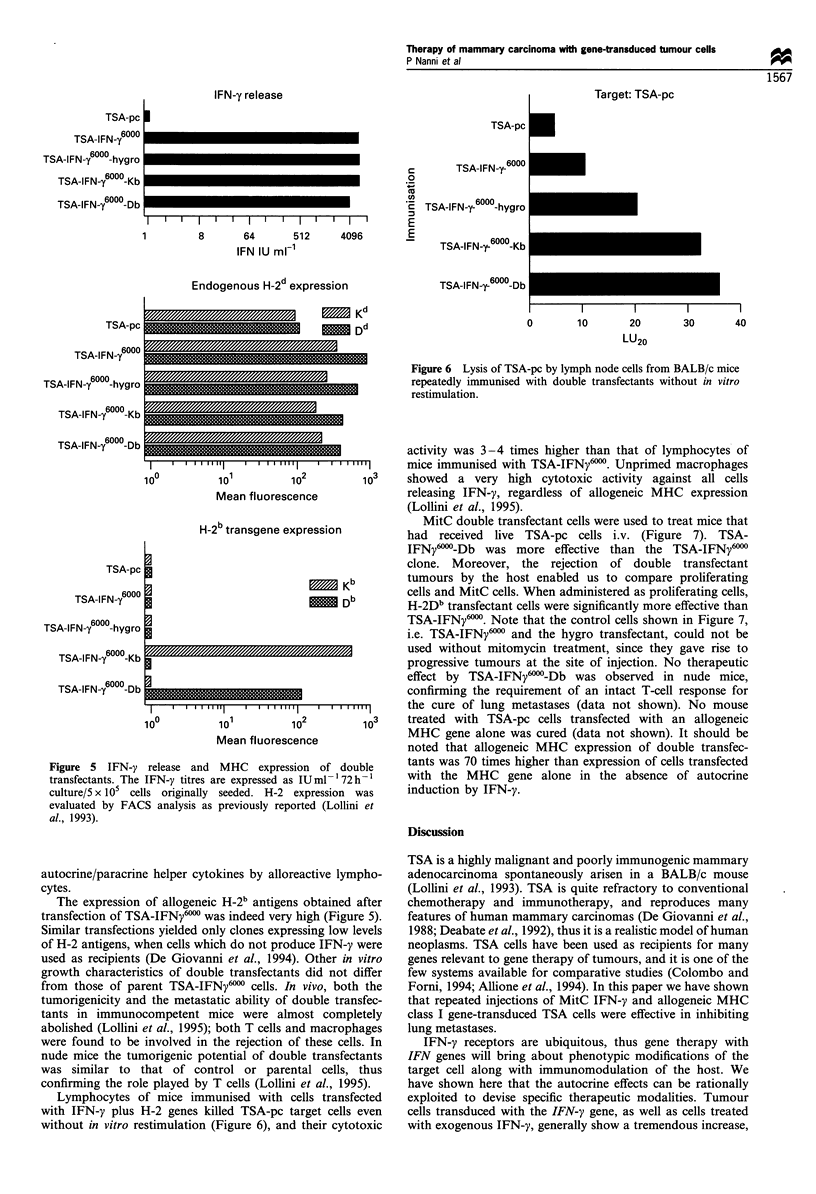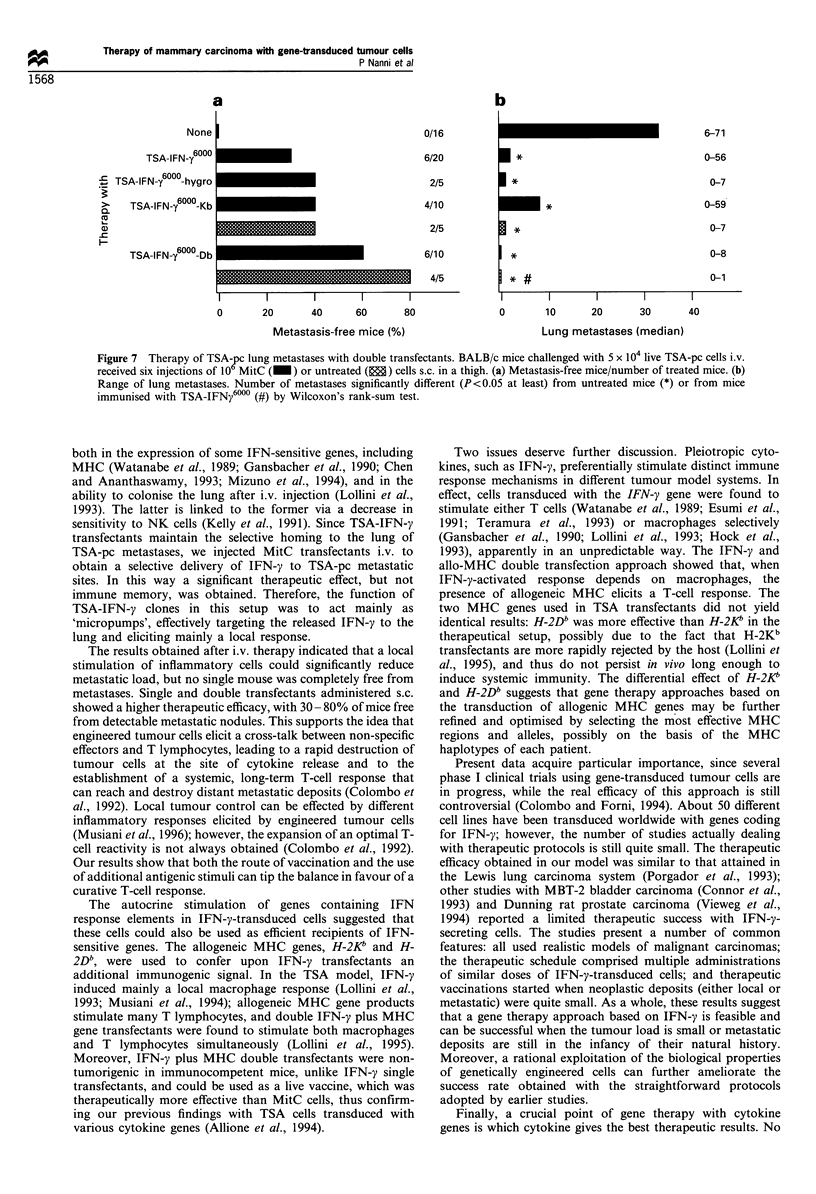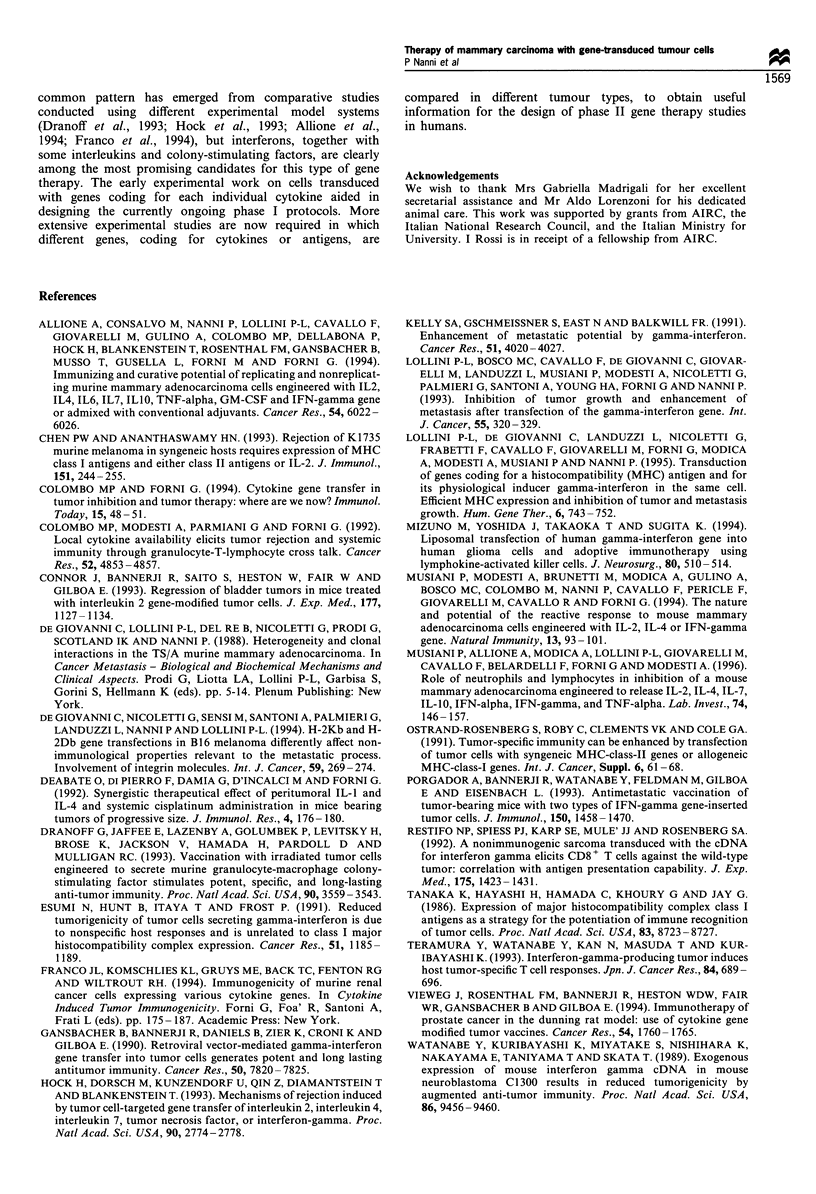# Therapy of murine mammary carcinoma metastasis with interferon gamma and MHC gene-transduced tumour cells.

**DOI:** 10.1038/bjc.1996.590

**Published:** 1996-11

**Authors:** P. Nanni, C. De Giovanni, L. Landuzzi, G. Nicoletti, F. Frabetti, I. Rossi, F. Cavallo, M. Giovarelli, G. Forni, P. L. Lollini

**Affiliations:** Istituto di Cancerologia, Universita di Bologna, Italy.

## Abstract

Gene-transfected tumour cells were used to cure mice bearing lung metastases by the parental, non-transduced mammary adenocarcinoma (TSA-pc). Repeated subcutaneous (s.c.) administrations of mitomycin C (MitC)-treated interferon gamma (IFN-gamma) transfectants induced a 90% inhibition in the number of lung metastases. Therapeutic effect required an intact T-cell response, as shown by the lack of efficacy in nude mice. Autocrine stimulation by IFN-gamma induces specific modifications in the phenotype of transfectants that acquire a high metastatic ability and show a high expression of IFN-responsive genes; these two features were exploited to design two experimental protocols to obtain an improvement of the therapeutic effect. The increased metastatic ability of IFN-gamma transfectants was used to deliver IFN-gamma selectively to the lungs of mice bearing TSA-pc pulmonary metastases. A significant therapeutic effect was obtained when TSA-pc experimental metastases were treated by repeated intravenous (i.v.) injections of MitC IFN-gamma transfectants. Since i.v. administrations of IFN-gamma transfectants did not induce immune memory, the therapeutical effect appeared to depend on the inflammatory-like response activated by local IFN release. To exploit the autocrine stimulation of IFN-sensitive genes an IFN-gamma transfectant clone was subjected to a second transfection with an allogeneic class I MHC gene (H-2K(b) or H-2D(h)). IFN-gamma plus MHC double transfectants maintained IFN-gamma release, showed a very high expression of the MHC gene products, stimulated both macrophages and T cells, and were less tumorigenic in immunocompetent mice than the parent IFN-gamma clone. Therapeutic efficacy of double transfectant IFN-gamma plus H-2D(b) cells against TSA-pc was superior to single transfectants, showing that the reaction elicited by genetically engineered cells can be selectively tuned to increase therapeutic efficacy.


British Journal of Cancer (1996) 74, 1564-1569
© 1996 Stockton Press All rights reserved 0007-0920/96 $12.00

### Therapy of murine mammary carcinoma metastasis with interferon γ and MHC gene-transduced tumour cells

P Nanni[1,2], C De Giovanni[1,2], L Landuzzi[1,3], G Nicoletti[1,3], F Frabetti[1], I Rossi[1], F Cavallo[4], M Giovarelli[4], G Forni[4,5] and P-L Lollini[1,2]

[1]*Istituto di Cancerologia, Università di Bologna;* [2]*Centro Interdipartimentale di Ricerche sul Cancro 'Giorgio Prodi', Università di Bologna;* [3]*IST, Sezione di Biotecnologie di Bologna;* [4]*Dipartimento di Scienze Cliniche e Biologiche, Università di Torino;* [5]*Centro CNR di Immunogenetica e Oncologia Sperimentale, Italy.*

**Summary** Gene-transfected tumour cells were used to cure mice bearing lung metastases by the parental, non-transduced mammary adenocarcinoma (TSA-pc). Repeated subcutaneous (s.c.) administrations of mitomycin C (MitC)-treated interferon γ (IFN-γ) transfectants induced a 90% inhibition in the number of lung metastases. Therapeutic effect required an intact T-cell response, as shown by the lack of efficacy in nude mice. Autocrine stimulation by IFN-γ induces specific modifications in the phenotype of transfectants that acquire a high metastatic ability and show a high expression of IFN-responsive genes; these two features were exploited to design two experimental protocols to obtain an improvement of the therapeutic effect. The increased metastatic ability of IFN-γ transfectants was used to deliver IFN-γ selectively to the lungs of mice bearing TSA-pc pulmonary metastases. A significant therapeutic effect was obtained when TSA-pc experimental metastases were treated by repeated intravenous (i.v.) injections of MitC IFN-γ transfectants. Since i.v. administrations of IFN-γ transfectants did not induce immune memory, the therapeutical effect appeared to depend on the inflammatory-like response activated by local IFN release. To exploit the autocrine stimulation of IFN-sensitive genes an IFN-γ transfectant clone was subjected to a second transfection with an allogeneic class I MHC gene (*H-2K^b^* or *H-2D^b^*). IFN-γ plus MHC double transfectants maintained IFN-γ release, showed a very high expression of the MHC gene products, stimulated both macrophages and T cells, and were less tumorigenic in immunocompetent mice than the parent IFN-γ clone. Therapeutic efficacy of double transfectant IFN-γ plus $H\text{-}2D^b$ cells against TSA-pc was superior to single transfectants, showing that the reaction elicited by genetically engineered cells can be selectively tuned to increase therapeutic efficacy.

**Keywords:** gene therapy; interferon gamma; major histocompatibility complex

The use of gene transduction has considerably expanded the range of tools available for the immunotherapy of tumours. Protection from tumour growth in preimmunised animals has been obtained with tumour cells transduced with genes for various cytokines and surface antigens (Tanaka *et al.*, 1986; Ostrand-Rosenberg *et al.*, 1991; Colombo and Forni, 1994). However, realistic therapeutic protocols, in which engineered cells are administered only after the challenge with live parent tumour cells, have been explored less frequently.

Malignant tumour cells transduced with foreign genes are a complex biological reagent. In fact, they should be regarded as a novel type of tumour, rather than as a passive immunological vaccine. In particular, some of the cytokines used for this type of studies are active both on the host immune system and on the tumour itself. In particular, interferon (IFN) gene transduction profoundly alters several properties of the transformed cells, including upmodulation of MHC, resistance to natural killer (NK) cells and metastatic ability (Watanabe *et al.*, 1989; Gansbacher *et al.*, 1990; Restifo *et al.*, 1992; Lollini *et al.*, 1993).

In principle, genes coding for class I major histocompatibility complex (MHC) should modify the interaction of tumour cells with the immune system only, but it has been clearly shown that after transfection of MHC genes a few properties of the neoplastic cells are also modified (De Giovanni *et al.*, 1994).

These considerations suggest that the biological properties of recipient tumour cells must be adequately characterised, before and after gene transfer, to exploit the possibilities offered by this technology fully. In this work we show how to take advantage for therapeutic purposes of some specific modifications induced by transfection of the IFN-γ gene in a mammary carcinoma.

### Materials and methods

### *Cells and DNA transfection*

TSA-pc is a tumour cell line we derived from a spontaneous mammary adenocarcinoma of the BALB/c strain; TSA-pc cells give rise to moderately differentiated, non-capsulated invasive tumours, which are highly metastatic and poorly immunogenic in syngeneic mice (Lollini *et al.*, 1993). TSA-pc has been used as the recipient of several cytokine genes (Colombo and Forni, 1994), including *IFN-γ* (Lollini *et al.*, 1993). TSA-IFN$\gamma^{500}$ and TSA-IFN$\gamma^{6000}$ were obtained from TSA following gene transfection with the murine *IFN-γ* gene and release 500 and 6000 IU $ml^{-1}$ of IFN-γ (Lollini *et al.*, 1993); TSA-neo control was transfected with the neomycin resistance gene alone (Lollini *et al.*, 1993). Clone TSA-IFN$\gamma^{6000}$ was subsequently transfected with allogeneic *$H\text{-}2K^b$* and *$H\text{-}2D^b$* genes (Lollini *et al.*, 1995) to obtain clones designated TSA-IFN$\gamma^{6000}$-Kb or TSA-IFN$\gamma^{6000}$-Db. Hygromycin resistance was used to select transfectants; clone TSA-IFN$\gamma^{6000}$-hygro was transfected with the hygromycin resistance gene alone. Cells were cultured in Dulbecco's modified eagle medium (DMEM; Gibco, Paisley, UK), supplemented with 10% heat-inactivated fetal bovine serum (FBS; Gibco); cultures were maintained at 37°C in a humidified atmosphere of 5% carbon dioxide in air. All the cells employed were >90% viable as judged by trypan blue dye exclusion.

### *Mice and TSA-pc challenge*

Seven-week-old female BALB/cAnNCrlBR (BALB/c) mice and 4-week-old female *nu/nu* mice on Swiss CD-1 back-

Correspondence: P Nanni, Istituto di Cancerologia, Viale Filopanti 22, I-40126 Bologna, Italy
Received 8 March 1996; revised 22 May 1996; accepted 31 May 1996

ground were purchased from Charles River Laboratories (Calco, Italy) and treated according to European Community guidelines. Mice were allowed to rest for 1 week before any treatment. Metastases were induced by i.v. injection of TSA-pc cells in a lateral tail vein ($5 \times 10^4$ cells in BALB/c mice or $5 \times 10^5$ cells in nude mice). Lung nodules were evaluated at day +21; all metastasis counts were performed on dissected lung lobes contrasted with black India ink under a stereoscopic microscope.

### *Treatment*

Either before or after the TSA-pc challenge, mice received transfectants treated with 60 $\mu g\ ml^{-1}$ mitomycin-C (Sigma, Milan, Italy) at 37°C for 45 min to abolish their residual tumorigenicity (Allione *et al.*, 1994). Prophylactic vaccinations consisted of a single injection of $1 \times 10^6$ mitomycin-C-treated (MitC) cells performed 30 days before challenge. Therapeutic vaccinations started 1 day after TSA-pc challenge and consisted of six injections of $1 \times 10^6$ MitC cells 3–4 days apart.

### In vitro *cytotoxicity tests*

Cytotoxicity was tested using lymph node (axillary, inguinal and mesenteric) cells or macrophages from mice repeatedly immunised with $1 \times 10^6$ MitC cells s.c. In some experiments, effectors were restimulated by a 6 day *in vitro* coculture with MitC TSA-pc cells and then tested for cytotoxic activity. Target cells were labelled by incubating non-confluent monolayers in 25 $cm^2$ tissue culture flasks for 24 h with 5 ml of medium containing 1 $\mu Ci\ ml^{-1}$ [$^3$H]thymidine (26 Ci $mmol^{-1}$; NEN, Milan, Italy). Target cells were then harvested by trypsin–EDTA (Gibco) treatment, washed twice and resuspended to a concentration of $5 \times 10^4$ cells $ml^{-1}$ in the test medium. Effector cells were admixed in triplicate with $5 \times 10^3$ labelled target cells at 50:1, 25:1, 12:1 and 6:1 E:T ratios in round-bottomed 96-well microtitre plates. After 48 h incubation at 37°C in 5% carbon dioxide, 0.1 ml of the supernatant was taken and the percentage of specific lysis was calculated as previously described (Lollini *et al.*, 1993). In some experiments, $^{51}$Cr-labelled TSA-pc cells were also used to evaluate 4 h release in the presence of effectors obtained as above, but very low values of cytotoxicity were obtained owing to the resistance to lysis of TSA-pc target cells (data not shown). Maximum release was determined by adding 0.1 ml of 10% Triton X-100. Values of spontaneous release were within 10–15%. The release values are expressed as lytic units (LU) calculated as described (Lollini *et al.*, 1993). One LU is here defined as the number of effector cells needed to kill 20% of the target cells ($LU_{20}$).

### *Statistical analysis*

The non-parametric Wilcoxon's rank-sum test was used to compare numbers of metastases.

### Results

### *Immunogenicity of TSA-IFNγ clones*

TSA-pc is a highly malignant tumour, and expression of the *IFN-γ* gene did not abolish completely the tumorigenicity of clones TSA-IFN$\gamma^{500}$ and TSA-IFN$\gamma^{6000}$ (which release 500 and 6000 IU $ml^{-1}$ IFN-γ respectively), thus mitomycin-C-treated (MitC) cells were used for therapeutic administrations. MitC cells retain for several days the ability to release IFN-γ, as shown by reverse transcriptase–polymerase chain reaction and IFN-γ bioassay (data not shown).

We have previously shown that the reduced tumorigenic potential of IFN-γ clones was mainly caused by the local activity of macrophages. However, tumour infiltrate also contained activated lymphocytes (Lollini *et al.*, 1993). IFN-γ clones induced memory cells directed against the parent tumour TSA-pc; mice immunised with IFN-γ clones were protected against a subsequent challenge with TSA-pc (Musiani *et al.*, 1994). Mice immunised with TSA-IFN$\gamma^{500}$ or TSA-IFN$\gamma^{6000}$ displayed a stronger cytotoxicity against TSA-pc than mice immunised with TSA-pc or TSA-neo (Figure 1).

**Figure 1** Lysis of TSA-pc by restimulated lymph node cells from BALB/c mice immunised s.c. in the right thigh with $10^6$ MitC cells.

**Figure 2** Therapy of TSA-pc lung metastases with IFN-γ transfectants. BALB/c mice challenged with $5 \times 10^4$ live TSA-pc cells i.v. received six injections of $10^6$ MitC cells s.c. in a thigh. (**a**) Metastasis-free mice/number of treated mice. (**b**) Range of lung metastases. *Number of metastases significantly different ($P<0.01$ at least) from untreated or TSA-pc-treated mice by Wilcoxon's rank-sum test.

*Therapy of lung metastases*

The ability of IFN-γ clones to enhance macrophage activity (Lollini *et al.*, 1993) and to elicit memory cells suggests that these clones could be used for immunotherapy. Since a major goal of cancer immunotherapy is to prevent the development of overt metastases in tumour-free hosts, BALB/c mice were first challenged i.v. with TSA-pc cells and then repeatedly treated s.c. with MitC TSA-IFNγ clones (Figure 2). Therapy with IFN-γ clones yielded more than one-third of mice free from lung metastases and reduced by 90% the median number of metastatic foci per mouse.

The lack of therapeutic efficacy of IFN-γ transfectant cells in nude mice (Figure 3), compared with immunocompetent hosts (see Figure 2), showed that an intact T-cell response is required for the cure of lung metastases.

*Self-targeting of transfectants*

The treatment of tumour cells with IFN-γ enhances their survival in the post-intravasation phases of the metastatic process, probably through an increase in resistance to NK cells (Kelly *et al.*, 1991). TSA-IFNγ$^{500}$ and TSA-IFNγ$^{6000}$, even though they have a reduced tumorigenic potential compared with TSA-pc, are more metastatic after i.v. injection (Lollini *et al.*, 1993). However, this higher metastatic capacity of TSA-IFNγ clones could be exploited for a selective delivery of IFN-γ-releasing cells to the lung, where TSA-pc metastasises.

Therefore, the therapeutic effect of IFN-γ clones administered i.v., a route which is not commonly used for immunotherapy in mice, was next evaluated. First, the immune memory against TSA-pc experimental metastasis elicited by MitC IFN-γ clones injected i.v. was evaluated. The preimmunisation (day −30) with either MitC TSA-pc or IFN-γ clones did not elicit protection against a TSA-pc i.v. challenge (day 0) (data not shown). By contrast, when MitC TSA-IFN-γ clones were administered i.v. after an i.v. TSA-pc challenge, a significant inhibition in the number of lung nodules was found (Figure 4). On the other hand, the same treatment with MitC TSA-pc actually enhanced the development of metastases. Taken together, these results suggest that the IFN-γ released by transfectants in the lungs acts via inflammatory, rather than memory, components.

*Therapy of lung colonies with IFN-γ plus H-2$^b$ double transfectants*

The results with IFN-γ clones were encouraging, especially considering that TSA is a highly malignant tumour. However, to potentiate further the interactions of tumour cells with the immune system of the host, we retransfected TSA-IFNγ$^{6000}$ cells with allogeneic *H-2K$^b$* or *H-2D$^b$* genes to exploit the high expression of IFN-induced molecules in TSA-IFN-γ clones. Moreover, since IFN-γ transfectants appeared to stimulate macrophages more than T cells, the addition of allogeneic MHC antigens could tip the balance in favour of a T response, mainly through the release of

**Figure 3** Lack of therapeutic effect on TSA-pc lung metastases in nude mice. Mice challenged with $5 \times 10^5$ live TSA-pc cells i.v. received six injections of $10^6$ MitC cells s.c. in a thigh. (**a**) Metastasis-free mice/number of treated mice. (**b**) Range of lung metastases.

**Figure 4** Therapy of TSA-pc lung metastases with IFN-γ transfectants administered i.v; BALB/c mice challenged with $5 \times 10^4$ live TSA-pc cells i.v. received six injections of $10^6$ MitC cells i.v. (**a**) Metastasis-free mice/number of treated mice. (**b**) Range of lung metastases. *Number of metastases significantly different ($P < 0.01$ at least) from untreated or TSA-pc-treated mice by Wilcoxon's rank-sum test.

**Figure 5** IFN-$\gamma$ release and MHC expression of double transfectants. The IFN-$\gamma$ titres are expressed as $IU ml^{-1} 72 h^{-1}$ culture/$5 \times 10^5$ cells originally seeded. H-2 expression was evaluated by FACS analysis as previously reported (Lollini *et al.*, 1993).

**Figure 6** Lysis of TSA-pc by lymph node cells from BALB/c mice repeatedly immunised with double transfectants without *in vitro* restimulation.

autocrine/paracrine helper cytokines by alloreactive lymphocytes.

The expression of allogeneic H-$2^b$ antigens obtained after transfection of TSA-IFN$\gamma^{6000}$ was indeed very high (Figure 5). Similar transfections yielded only clones expressing low levels of H-2 antigens, when cells which do not produce IFN-$\gamma$ were used as recipients (De Giovanni *et al.*, 1994). Other *in vitro* growth characteristics of double transfectants did not differ from those of parent TSA-IFN$\gamma^{6000}$ cells. *In vivo*, both the tumorigenicity and the metastatic ability of double transfectants in immunocompetent mice were almost completely abolished (Lollini *et al.*, 1995); both T cells and macrophages were found to be involved in the rejection of these cells. In nude mice the tumorigenic potential of double transfectants was similar to that of control or parental cells, thus confirming the role played by T cells (Lollini *et al.*, 1995).

Lymphocytes of mice immunised with cells transfected with IFN-$\gamma$ plus H-2 genes killed TSA-pc target cells even without *in vitro* restimulation (Figure 6), and their cytotoxic activity was 3–4 times higher than that of lymphocytes of mice immunised with TSA-IFN$\gamma^{6000}$. Unprimed macrophages showed a very high cytotoxic activity against all cells releasing IFN-$\gamma$, regardless of allogeneic MHC expression (Lollini *et al.*, 1995).

MitC double transfectant cells were used to treat mice that had received live TSA-pc cells i.v. (Figure 7). TSA-IFN$\gamma^{6000}$-Db was more effective than the TSA-IFN$\gamma^{6000}$ clone. Moreover, the rejection of double transfectant tumours by the host enabled us to compare proliferating cells and MitC cells. When administered as proliferating cells, H-2$D^b$ transfectant cells were significantly more effective than TSA-IFN$\gamma^{6000}$. Note that the control cells shown in Figure 7, i.e. TSA-IFN$\gamma^{6000}$ and the hygro transfectant, could not be used without mitomycin treatment, since they gave rise to progressive tumours at the site of injection. No therapeutic effect by TSA-IFN$\gamma^{6000}$-Db was observed in nude mice, confirming the requirement of an intact T-cell response for the cure of lung metastases (data not shown). No mouse treated with TSA-pc cells transfected with an allogeneic MHC gene alone was cured (data not shown). It should be noted that allogeneic MHC expression of double transfectants was 70 times higher than expression of cells transfected with the MHC gene alone in the absence of autocrine induction by IFN-$\gamma$.

### Discussion

TSA is a highly malignant and poorly immunogenic mammary adenocarcinoma spontaneously arisen in a BALB/c mouse (Lollini *et al.*, 1993). TSA is quite refractory to conventional chemotherapy and immunotherapy, and reproduces many features of human mammary carcinomas (De Giovanni *et al.*, 1988; Deabate *et al.*, 1992), thus it is a realistic model of human neoplasms. TSA cells have been used as recipients for many genes relevant to gene therapy of tumours, and it is one of the few systems available for comparative studies (Colombo and Forni, 1994; Allione *et al.*, 1994). In this paper we have shown that repeated injections of MitC IFN-$\gamma$ and allogeneic MHC class I gene-transduced TSA cells were effective in inhibiting lung metastases.

IFN-$\gamma$ receptors are ubiquitous, thus gene therapy with *IFN* genes will bring about phenotypic modifications of the target cell along with immunomodulation of the host. We have shown here that the autocrine effects can be rationally exploited to devise specific therapeutic modalities. Tumour cells transduced with the *IFN-$\gamma$* gene, as well as cells treated with exogenous IFN-$\gamma$, generally show a tremendous increase,

**Figure 7** Therapy of TSA-pc lung metastases with double transfectants. BALB/c mice challenged with $5 \times 10^4$ live TSA-pc cells i.v. received six injections of $10^6$ MitC (■) or untreated (▩) cells s.c. in a thigh. (**a**) Metastasis-free mice/number of treated mice. (**b**) Range of lung metastases. Number of metastases significantly different ($P<0.05$ at least) from untreated mice (*) or from mice immunised with TSA-IFN$\gamma^{6000}$ (#) by Wilcoxon's rank-sum test.

both in the expression of some IFN-sensitive genes, including MHC (Watanabe *et al.*, 1989; Gansbacher *et al.*, 1990; Chen and Ananthaswamy, 1993; Mizuno *et al.*, 1994), and in the ability to colonise the lung after i.v. injection (Lollini *et al.*, 1993). The latter is linked to the former via a decrease in sensitivity to NK cells (Kelly *et al.*, 1991). Since TSA-IFN-γ transfectants maintain the selective homing to the lung of TSA-pc metastases, we injected MitC transfectants i.v. to obtain a selective delivery of IFN-γ to TSA-pc metastatic sites. In this way a significant therapeutic effect, but not immune memory, was obtained. Therefore, the function of TSA-IFN-γ clones in this setup was to act mainly as 'micropumps', effectively targeting the released IFN-γ to the lung and eliciting mainly a local response.

The results obtained after i.v. therapy indicated that a local stimulation of inflammatory cells could significantly reduce metastatic load, but no single mouse was completely free from metastases. Single and double transfectants administered s.c. showed a higher therapeutic efficacy, with 30–80% of mice free from detectable metastatic nodules. This supports the idea that engineered tumour cells elicit a cross-talk between non-specific effectors and T lymphocytes, leading to a rapid destruction of tumour cells at the site of cytokine release and to the establishment of a systemic, long-term T-cell response that can reach and destroy distant metastatic deposits (Colombo *et al.*, 1992). Local tumour control can be effected by different inflammatory responses elicited by engineered tumour cells (Musiani *et al.*, 1996); however, the expansion of an optimal T-cell reactivity is not always obtained (Colombo *et al.*, 1992). Our results show that both the route of vaccination and the use of additional antigenic stimuli can tip the balance in favour of a curative T-cell response.

The autocrine stimulation of genes containing IFN response elements in IFN-γ-transduced cells suggested that these cells could also be used as efficient recipients of IFN-sensitive genes. The allogeneic MHC genes, *H-2K^b^* and *H-2D^b^*, were used to confer upon IFN-γ transfectants an additional immunogenic signal. In the TSA model, IFN-γ induced mainly a local macrophage response (Lollini *et al.*, 1993; Musiani *et al.*, 1994); allogeneic MHC gene products stimulate many T lymphocytes, and double IFN-γ plus MHC gene transfectants were found to stimulate both macrophages and T lymphocytes simultaneously (Lollini *et al.*, 1995). Moreover, IFN-γ plus MHC double transfectants were non-tumorigenic in immunocompetent mice, unlike IFN-γ single transfectants, and could be used as a live vaccine, which was therapeutically more effective than MitC cells, thus confirming our previous findings with TSA cells transduced with various cytokine genes (Allione *et al.*, 1994).

Two issues deserve further discussion. Pleiotropic cytokines, such as IFN-γ, preferentially stimulate distinct immune response mechanisms in different tumour model systems. In effect, cells transduced with the *IFN-γ* gene were found to stimulate either T cells (Watanabe *et al.*, 1989; Esumi *et al.*, 1991; Teramura *et al.*, 1993) or macrophages selectively (Gansbacher *et al.*, 1990; Lollini *et al.*, 1993; Hock *et al.*, 1993), apparently in an unpredictable way. The IFN-γ and allo-MHC double transfection approach showed that, when IFN-γ-activated response depends on macrophages, the presence of allogeneic MHC elicits a T-cell response. The two MHC genes used in TSA transfectants did not yield identical results: $H\text{-}2D^b$ was more effective than $H\text{-}2K^b$ in the therapeutical setup, possibly due to the fact that $\text{H-2K}^b$ transfectants are more rapidly rejected by the host (Lollini *et al.*, 1995), and thus do not persist *in vivo* long enough to induce systemic immunity. The differential effect of $H\text{-}2K^b$ and $H\text{-}2D^b$ suggests that gene therapy approaches based on the transduction of allogenic MHC genes may be further refined and optimised by selecting the most effective MHC regions and alleles, possibly on the basis of the MHC haplotypes of each patient.

Present data acquire particular importance, since several phase I clinical trials using gene-transduced tumour cells are in progress, while the real efficacy of this approach is still controversial (Colombo and Forni, 1994). About 50 different cell lines have been transduced worldwide with genes coding for IFN-γ; however, the number of studies actually dealing with therapeutic protocols is still quite small. The therapeutic efficacy obtained in our model was similar to that attained in the Lewis lung carcinoma system (Porgador *et al.*, 1993); other studies with MBT-2 bladder carcinoma (Connor *et al.*, 1993) and Dunning rat prostate carcinoma (Vieweg *et al.*, 1994) reported a limited therapeutic success with IFN-γ-secreting cells. The studies present a number of common features: all used realistic models of malignant carcinomas; the therapeutic schedule comprised multiple administrations of similar doses of IFN-γ-transduced cells; and therapeutic vaccinations started when neoplastic deposits (either local or metastatic) were quite small. As a whole, these results suggest that a gene therapy approach based on IFN-γ is feasible and can be successful when the tumour load is small or metastatic deposits are still in the infancy of their natural history. Moreover, a rational exploitation of the biological properties of genetically engineered cells can further ameliorate the success rate obtained with the straightforward protocols adopted by earlier studies.

Finally, a crucial point of gene therapy with cytokine genes is which cytokine gives the best therapeutic results. No common pattern has emerged from comparative studies conducted using different experimental model systems (Dranoff *et al.*, 1993; Hock *et al.*, 1993; Allione *et al.*, 1994; Franco *et al.*, 1994), but interferons, together with some interleukins and colony-stimulating factors, are clearly among the most promising candidates for this type of gene therapy. The early experimental work on cells transduced with genes coding for each individual cytokine aided in designing the currently ongoing phase I protocols. More extensive experimental studies are now required in which different genes, coding for cytokines or antigens, are compared in different tumour types, to obtain useful information for the design of phase II gene therapy studies in humans.

**Acknowledgements**
We wish to thank Mrs Gabriella Madrigali for her excellent secretarial assistance and Mr Aldo Lorenzoni for his dedicated animal care. This work was supported by grants from AIRC, the Italian National Research Council, and the Italian Ministry for University. I Rossi is in receipt of a fellowship from AIRC.

### References

ALLIONE A, CONSALVO M, NANNI P, LOLLINI P-L, CAVALLO F, GIOVARELLI M, GULINO A, COLOMBO MP, DELLABONA P, HOCK H, BLANKENSTEIN T, ROSENTHAL FM, GANSBACHER B, MUSSO T, GUSELLA L, FORNI M AND FORNI G. (1994). Immunizing and curative potential of replicating and nonreplicating murine mammary adenocarcinoma cells engineered with IL2, IL4, IL6, IL7, IL10, TNF-alpha, GM-CSF and IFN-gamma gene or admixed with conventional adjuvants. *Cancer Res.*, **54**, 6022–6026.

CHEN PW AND ANANTHASWAMY HN. (1993). Rejection of K1735 murine melanoma in syngeneic hosts requires expression of MHC class I antigens and either class II antigens or IL-2. *J. Immunol.*, **151**, 244–255.

COLOMBO MP AND FORNI G. (1994). Cytokine gene transfer in tumor inhibition and tumor therapy: where are we now? *Immunol. Today*, **15**, 48–51.

COLOMBO MP, MODESTI A, PARMIANI G AND FORNI G. (1992). Local cytokine availability elicits tumor rejection and systemic immunity through granulocyte-T-lymphocyte cross talk. *Cancer Res.*, **52**, 4853–4857.

CONNOR J, BANNERJI R, SAITO S, HESTON W, FAIR W AND GILBOA E. (1993). Regression of bladder tumors in mice treated with interleukin 2 gene-modified tumor cells. *J. Exp. Med.*, **177**, 1127–1134.

DE GIOVANNI C, LOLLINI P-L, DEL RE B, NICOLETTI G, PRODI G, SCOTLAND IK AND NANNI P. (1988). Heterogeneity and clonal interactions in the TS/A murine mammary adenocarcinoma. In *Cancer Metastasis – Biological and Biochemical Mechanisms and Clinical Aspects*. Prodi G, Liotta LA, Lollini P-L, Garbisa S, Gorini S, Hellmann K (eds). pp. 5-14. Plenum Publishing: New York.

DE GIOVANNI C, NICOLETTI G, SENSI M, SANTONI A, PALMIERI G, LANDUZZI L, NANNI P AND LOLLINI P-L. (1994). H-2Kb and H-2Db gene transfections in B16 melanoma differently affect non-immunological properties relevant to the metastatic process. Involvement of integrin molecules. *Int. J. Cancer*, **59**, 269–274.

DEABATE O, DI PIERRO F, DAMIA G, D'INCALCI M AND FORNI G. (1992). Synergistic therapeutical effect of peritumoral IL-1 and IL-4 and systemic cisplatinum administration in mice bearing tumors of progressive size. *J. Immunol. Res.*, **4**, 176–180.

DRANOFF G, JAFFEE E, LAZENBY A, GOLUMBEK P, LEVITSKY H, BROSE K, JACKSON V, HAMADA H, PARDOLL D AND MULLIGAN RC. (1993). Vaccination with irradiated tumor cells engineered to secrete murine granulocyte-macrophage colony-stimulating factor stimulates potent, specific, and long-lasting anti-tumor immunity. *Proc. Natl Acad. Sci. USA*, **90**, 3559–3543.

ESUMI N, HUNT B, ITAYA T AND FROST P. (1991). Reduced tumorigenicity of tumor cells secreting gamma-interferon is due to nonspecific host responses and is unrelated to class I major histocompatibility complex expression. *Cancer Res.*, **51**, 1185–1189.

FRANCO JL, KOMSCHLIES KL, GRUYS ME, BACK TC, FENTON RG AND WILTROUT RH. (1994). Immunogenicity of murine renal cancer cells expressing various cytokine genes. In *Cytokine Induced Tumor Immunogenicity*. Forni G, Foa' R, Santoni A, Frati L (eds). pp. 175–187. Academic Press: New York.

GANSBACHER B, BANNERJI R, DANIELS B, ZIER K, CRONI K AND GILBOA E. (1990). Retroviral vector-mediated gamma-interferon gene transfer into tumor cells generates potent and long lasting antitumor immunity. *Cancer Res.*, **50**, 7820–7825.

HOCK H, DORSCH M, KUNZENDORF U, QIN Z, DIAMANTSTEIN T AND BLANKENSTEIN T. (1993). Mechanisms of rejection induced by tumor cell-targeted gene transfer of interleukin 2, interleukin 4, interleukin 7, tumor necrosis factor, or interferon-gamma. *Proc. Natl Acad. Sci. USA*, **90**, 2774–2778.

KELLY SA, GSCHMEISSNER S, EAST N AND BALKWILL FR. (1991). Enhancement of metastatic potential by gamma-interferon. *Cancer Res.*, **51**, 4020–4027.

LOLLINI P-L, BOSCO MC, CAVALLO F, DE GIOVANNI C, GIOVARELLI M, LANDUZZI L, MUSIANI P, MODESTI A, NICOLETTI G, PALMIERI G, SANTONI A, YOUNG HA, FORNI G AND NANNI P. (1993). Inhibition of tumor growth and enhancement of metastasis after transfection of the gamma-interferon gene. *Int. J. Cancer*, **55**, 320–329.

LOLLINI P-L, DE GIOVANNI C, LANDUZZI L, NICOLETTI G, FRABETTI F, CAVALLO F, GIOVARELLI M, FORNI G, MODICA A, MODESTI A, MUSIANI P AND NANNI P. (1995). Transduction of genes coding for a histocompatibility (MHC) antigen and for its physiological inducer gamma-interferon in the same cell. Efficient MHC expression and inhibition of tumor and metastasis growth. *Hum. Gene Ther.*, **6**, 743–752.

MIZUNO M, YOSHIDA J, TAKAOKA T AND SUGITA K. (1994). Liposomal transfection of human gamma-interferon gene into human glioma cells and adoptive immunotherapy using lymphokine-activated killer cells. *J. Neurosurg.*, **80**, 510–514.

MUSIANI P, MODESTI A, BRUNETTI M, MODICA A, GULINO A, BOSCO MC, COLOMBO M, NANNI P, CAVALLO F, PERICLE F, GIOVARELLI M, CAVALLO R AND FORNI G. (1994). The nature and potential of the reactive response to mouse mammary adenocarcinoma cells engineered with IL-2, IL-4 or IFN-gamma gene. *Natural Immunity*, **13**, 93–101.

MUSIANI P, ALLIONE A, MODICA A, LOLLINI P-L, GIOVARELLI M, CAVALLO F, BELARDELLI F, FORNI G AND MODESTI A. (1996). Role of neutrophils and lymphocytes in inhibition of a mouse mammary adenocarcinoma engineered to release IL-2, IL-4, IL-7, IL-10, IFN-alpha, IFN-gamma, and TNF-alpha. *Lab. Invest.*, **74**, 146–157.

OSTRAND-ROSENBERG S, ROBY C, CLEMENTS VK AND COLE GA. (1991). Tumor-specific immunity can be enhanced by transfection of tumor cells with syngeneic MHC-class-II genes or allogeneic MHC-class-I genes. *Int. J. Cancer*, **Suppl. 6**, 61–68.

PORGADOR A, BANNERJI R, WATANABE Y, FELDMAN M, GILBOA E AND EISENBACH L. (1993). Antimetastatic vaccination of tumor-bearing mice with two types of IFN-gamma gene-inserted tumor cells. *J. Immunol.*, **150**, 1458–1470.

RESTIFO NP, SPIESS PJ, KARP SE, MULE' JJ AND ROSENBERG SA. (1992). A nonimmunogenic sarcoma transduced with the cDNA for interferon gamma elicits $CD8^+$ T cells against the wild-type tumor: correlation with antigen presentation capability. *J. Exp. Med.*, **175**, 1423–1431.

TANAKA K, HAYASHI H, HAMADA C, KHOURY G AND JAY G. (1986). Expression of major histocompatibility complex class I antigens as a strategy for the potentiation of immune recognition of tumor cells. *Proc. Natl Acad. Sci. USA*, **83**, 8723–8727.

TERAMURA Y, WATANABE Y, KAN N, MASUDA T AND KURIBAYASHI K. (1993). Interferon-gamma-producing tumor induces host tumor-specific T cell responses. *Jpn. J. Cancer Res.*, **84**, 689–696.

VIEWEG J, ROSENTHAL FM, BANNERJI R, HESTON WDW, FAIR WR, GANSBACHER B AND GILBOA E. (1994). Immunotherapy of prostate cancer in the dunning rat model: use of cytokine gene modified tumor vaccines. *Cancer Res.*, **54**, 1760–1765.

WATANABE Y, KURIBAYASHI K, MIYATAKE S, NISHIHARA K, NAKAYAMA E, TANIYAMA T AND SKATA T. (1989). Exogenous expression of mouse interferon gamma cDNA in mouse neuroblastoma C1300 results in reduced tumorigenicity by augmented anti-tumor immunity. *Proc. Natl Acad. Sci. USA*, **86**, 9456–9460.